# Durable remission with ruxolitinib in a chronic neutrophilic leukemia patient harboring a truncation and membrane proximal *CSF3R* compound mutation

**DOI:** 10.1007/s00277-020-04152-w

**Published:** 2020-06-23

**Authors:** Anna Hinze, Jenny Rinke, Andreas Hochhaus, Thomas Ernst

**Affiliations:** Abteilung Hämatologie und Internistische Onkologie, Klinik für Innere Medizin II, Universitätsklinikum Jena, Am Klinikum 1, 07747 Jena, Germany

Dear Editor,

Chronic neutrophilic leukemia (CNL) is an extremely rare myeloproliferative disease. The World Health Organization (WHO) diagnostic criteria include peripheral blood leukocytes ≥ 25/nl, hypercellular bone marrow, not meeting the WHO criteria for *BCR-ABL1*^*+*^ chronic myeloid leukemia (CML), primary myelofibrosis, polycythemia vera and essential thrombocythemia, and no rearrangement of *PDGFRA*, *PDGFRB*, *FGFR1*, or *PCM1-JAK2*. Symptoms include splenomegaly, fatigue, weight loss, bruising, bone pain, and night sweats [1].

The latest revision of the WHO also includes somatic activating mutations in the colony-stimulating factor 3 receptor (*CSF3R*) gene as an additional criterion which supports the diagnosis of CNL [1]. However, these mutations appear to be very rare in atypical CML (aCML, < 10%), which is similarly characterized by neutrophilia. Hence, they can serve as a useful marker to differentiate these entities. Mutations in *CSF3R* occur as point mutations in the membrane proximal domain with increased receptor activity in the absence of the ligand, or as nonsense mutations which lead to a premature truncation of the cytoplasmic tail, causing constitutive receptor overexpression and ligand hypersensitivity [2]. In vitro studies have shown that truncation mutations preferentially activate downstream activating mediators like SRC family kinases and tyrosine kinase non-receptor 2 (TNK2), sensitizing cells to tyrosine kinase inhibitors such as dasatinib; for membrane proximal mutations, the JAK-STAT pathway is the dominant way of signaling causing sensitivity to JAK kinase inhibitors, like ruxolitinib [2]. The most commonly described mutation is the proximal membrane point mutation T618I, present in 88% of described CNL cases. It has been demonstrated that additional mutations in the *SETBP1* and *ASXL1* gene have poorer outcome due to lack of effectiveness of ruxolitinib [1].

**Fig. 1:**
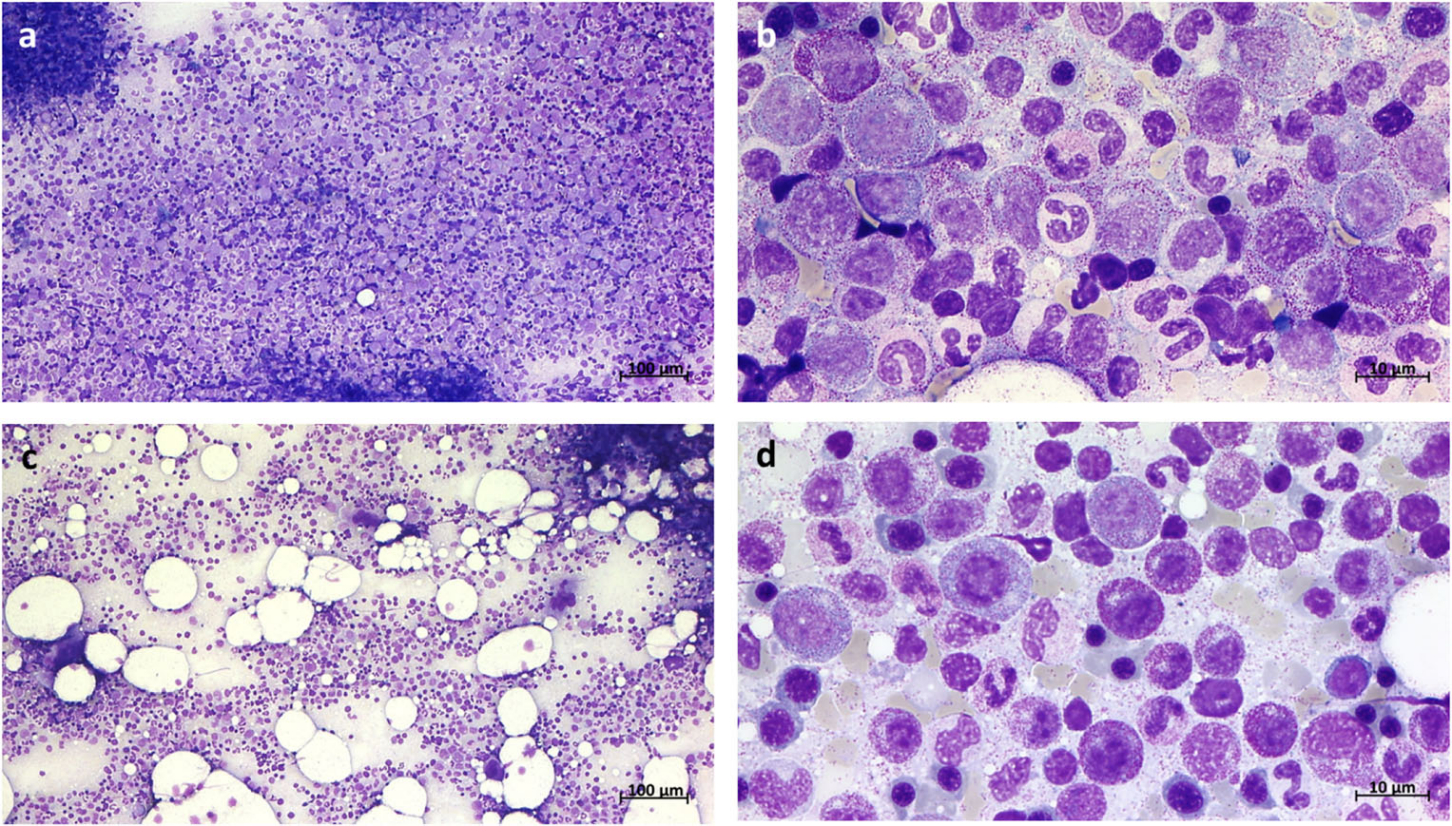
Pappenheim staining of bone marrow smears for the patient at time of diagnosis and under therapy with ruxolitinib. **a** and **b** Bone marrow biopsy at time of diagnosis in December 2016 showed a hypercellular marrow with an increased left-shifted granulopoiesis without blast proliferation. **c** and **d** The bone marrow biopsy of April 2018 showed an normocellular marrow with a sporadical left shift

**Fig. 2:**
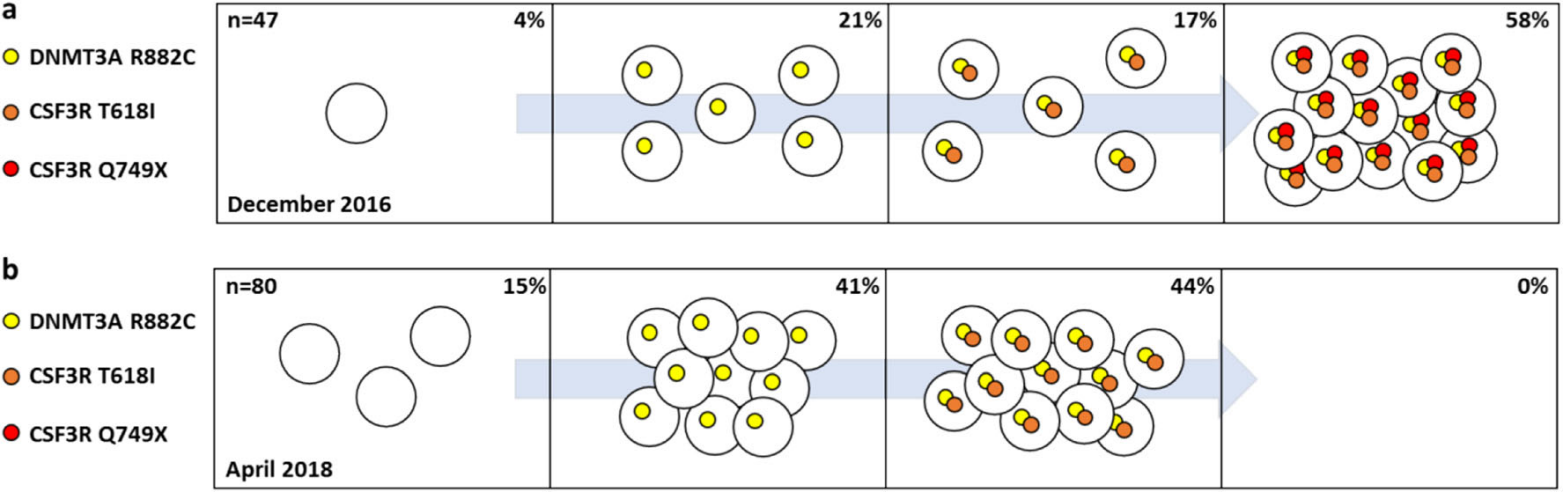
Clonal evolution of the *CSF3R* and *DNMT3A* mutations in the CNL patient. Targeted next-generation sequencing for myeloid malignancies (454 sequencing, GS Junior platform, Roche Diagnostics, Rotkreuz, Switzerland; TruSight Myeloid Panel, MiniSeq, Illumina, San Diego, CA, USA) revealed mutations in *CSF3R* (T618I, Q749X) and in *DNMT3A* (R882C). Individual colony-forming unit-granulocyte monocyte (CFU-GM) derived colonies from MNCs were isolated. Subsequently, a whole-genome amplification and pyrosequencing (PyroMark Q96, Qiagen, Hilden, Germany) was performed to analyze the clonal status of each colony. **a** In December 2016, a colony assay was performed, and colonies were picked and analyzed in respect to the T618I and Q749X mutations in the *CSF3R* gene and the R882C mutation in the *DNMT3A* gene. In total 47 colonies were analyzed and 4% of these colonies showed a wild type (2/47); 21% were heterozygous for the *DNMT3A* mutation (10/47); 17% were heterozygous for the *DNMT3A* (8/47) and *CSF3R* T618I mutation; and 58% harbored all three mutations (27/47). **b** At a second time point in April 2018 after more than 1 year of treatment with ruxolitinib, another colony assay was carried out. Of the analyzed colonies, 15% were wild type (12/80), and 41% harbored only the heterozygous *DNMT3A* mutation (33/80). The remaining 44% of colonies were heterozygous for the *DNMT3A* R882C and the *CSF3R* T618I point mutations (35/80). At this time point, no colony had a Q749X truncation mutation

**Fig. 3:**
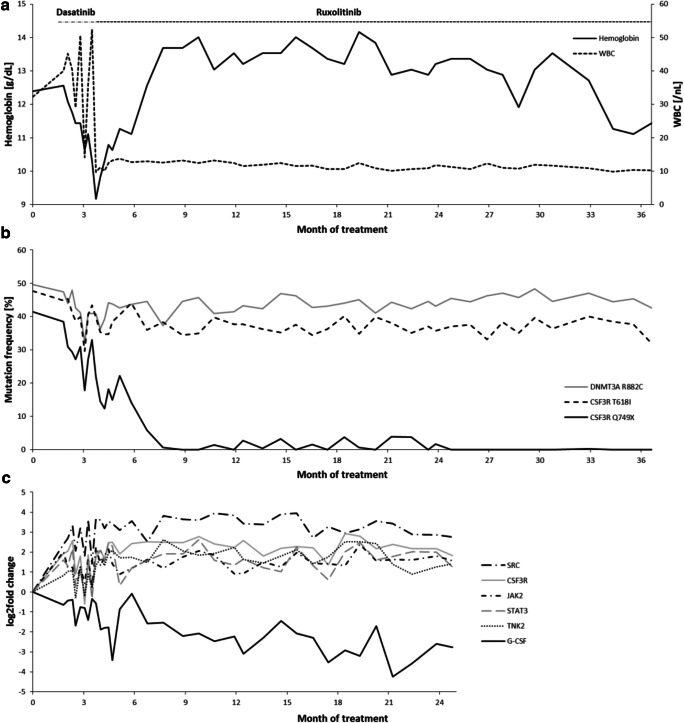
Blood counts, mutation frequency, and expression analyses. **a** At the beginning, the patient showed leukocytosis and a weak anemia. Under dasatinib treatment (day 54), the anemia got worse, and it led to an unstable WBC. Change to ruxolitinib (day 105) led to improved hemoglobin and WBC values. **b** Mutation frequency measured with pyrosequencing in follow-up samples over a period of 1099 days (~ 3 years). Samples have been measured at least once per month, and the last measurement was performed on day 1099. On day 0, the mutation frequencies were 47.7% for *CSF3R* T618I, 41.5% for *CSF3R* Q749X, and 49.7% for the *DNMT3A* mutation. The start of the treatment with dasatinib was on day 54 leading first to a decline of the mutation frequencies for all three mutations but then to a rapid growth. Dasatinib was discontinued, and ruxolitinib was commenced on day 105 resulting in a significant suppression of the clone harboring the *CSF3R* Q749X mutation, a reduction of the clone harboring *CSF3R* T618I of around 10%, and to a minor reduction of the *DNMT3A* R882C mutation frequency. **c** RT-PCR was performed to monitor the expression of *CSF3R*, the CSF3R-stimulating *G-CSF*, and downstream activating mediators for the JAK-STAT signaling pathway (*STAT3*, *JAK2*) and the SFK-TNK2 pathway (*SRC*, *TNK2*). *GUSB* was used as housekeeping gene. Data was analyzed with the ΔΔCT method and presented as log2fold change. During therapy with ruxolitinib, all analyzed genes increased in expression level except G-CSF which showed a lowering in expression level

Here we report a case of a 71-year-old man who first presented in November 2016 for the investigation of leukocytosis > 30/nl with a left shift and splenomegaly. His bone marrow biopsy showed a hypercellular marrow with an increased left-shifted granulopoiesis without blast proliferation (Fig. 1a, b). His complete blood count (CBC) showed a white blood cell count (WBC) 32.2/nl, hemoglobin (Hgb) 12.4 g/dl, and platelets 133/nl. CML was excluded by molecular testing and mutation screening for *JAK2* V617F was negative. Targeted next-generation sequencing revealed two *CSF3R* mutations, the common proximal membrane mutation T618I and a truncation mutation Q749X. In addition, the *DNMT3A* hotspot mutation R882C was detected. The patient was negative for mutations in *SETBP1* and *ASXL1*. Thus, diagnosis of CNL was made in December 2016. Dasatinib 100 mg/day was started according to recommendations from in vitro studies [2]. First his CBC improved (WBC 14.6/nl, Hgb 10.6 g/dl, platelets 89/nl). However, after 2 months of dasatinib therapy, the patient relapsed (WBC 50.6/nl, Hgb 10.3 g/dl, platelets 178/nl). A progenitor colony assay with mononuclear cells (MNCs) was performed indicating a clonal architecture of the mutations with the *DNMT3A* R882C mutation acquired first, followed by *CSF3R* T618I as the second and Q749X as the third mutation (Fig. 2a). According to these results and the experiments of Maxson et al. [2], the therapy was changed to 10 mg ruxolitinib twice a day resulting in a rapid reduction of leukocytes and to a prolonged hematologic remission (Fig. 3a) but not to a reduction of the allele burden of T618I. The mutations were monitored at least once a month for 3 years from November 2016 to November 2019. The results showed that *CSF3R* Q749X was suppressed over time, while the *CSF3R* T618I and the *DNMT3A* R882C mutation were only slightly reduced (Fig. 3b). After 1 year of treatment with ruxolitinib, a second progenitor colony assay was performed and showed that cell clones carrying the truncation mutation were eliminated, but the *DNMT3A* R882C and *CSF3R* T618I clones continued to be present (Fig. 2b). Expression analysis of downstream activating mediators (*SRC*, *TNK2*, *JAK2*, *STAT3*) of the *CSF3R* pathway showed a rise in expression levels and a decline for the CSF3R-stimulating molecule *G-CSF* (Fig. 3c). A bone marrow biopsy taken in April 2018 showed an normocellular marrow with a sporadical left shift (Fig. 1c, d). Data from November 2019 showed a normalization of the WBC (10.2/nl), Hgb (11.4 g/dl), and platelets (271/nl) and a normalization of the spleen size. Treatment with ruxolitinib was well tolerated without any non-hematological side effects.

The treatment with dasatinib was ineffective, leading only to a temporary reduction of the mutant clones. The effect of dasatinib on CNL patients with a truncation mutation is based on in vitro studies, and it remains unclear if this drug is effective on compound mutations in vivo. In the context of an additional proximal membrane mutation, dasatinib may not be sufficient. Therapy with ruxolitinib was successful leading to the suppression of the truncation mutation. In vitro studies showed that truncation mutations are also sensitive to ruxolitinib in case of exogenous G-CSF stimulation activating JAK-STAT signaling [2]. Perhaps in vivo the endogenous production of G-CSF leads to a stimulation of JAK-STAT signaling resulting in ruxolitinib sensitivity for the truncation mutation. It has previously been shown that the expression of a truncated *CSF3R* leads to hyperproliferation and hypersensitivity to G-CSF [3]. Hence, the suppression of the truncation mutation leads to an improvement of the clinical status of the patient and a normalization of hematopoiesis. Furthermore, the reduction of the endogenous G-CSF expression correlates with the improvement of the health status of the patient. The clones harboring both *CSF3R* mutations drive the clinical phenotype and are sensitive to the ruxolitinib treatment, while clones only harboring the T618I mutation survive. Thus, it is possible that the synergistic effect of both mutations causes a higher sensitivity to ruxolitinib. Still, it remains unclear why the clone only harboring the ruxolitinib-sensitive T618I mutation survives the treatment.

Most CNL cases described in the literature harbor the common T618I mutation with ruxolitinib used as a treatment option. Often the T618I mutation burden is initially reduced on therapy, but patients may relapse which is often based on additional mutations affecting *SETBP1* and *ASXL1* [4–6]. Recently, results of a clinical phase II trial were published investigating the safety and efficacy of ruxolitinib in 44 CNL and aCML patients irrespective of the *CSF3R* mutation status [7]. Ruxolitinib was well tolerated and demonstrated an estimated response rate of 32%. CNL patients harboring *CSF3R* T618I mutations were most likely to respond.

The detected *DNMT3A* R882C mutation is a hotspot mutation commonly mutated in acute myeloid leukemia (AML) patients and highly associated with poor prognosis [8]. *DNMT3A* mutations including the R882 mutation are also known to be preleukemic events [9]. *DNMT3A* mutations have not been described in CNL; hence, their effects on this leukemia entity are unknown. Although in the case presented here, the mutation was initially acquired with the *CSF3R* mutations as later events, the *DNMT3A* mutation does not seem to have an effect on the clinical status. Despite harboring a proximal membrane *CSF3R* mutation and a *DNMT3A* hotspot mutation, the patient remains in remission for > 3 years. Nevertheless, our CNL patient needs to be continuously monitored since he has a high risk of relapse and progression to secondary AML.

The presented case showed that dasatinib therapy in the case of a *CSF3R* compound mutation consisting of a primary proximal membrane point mutation (T618I) and a secondary truncation mutation (Q749X) is ineffective. The therapy of choice in this case was the JAK1/2 inhibitor ruxolitinib.
